# Housing Temperature Modulates the Impact of Diet-Induced Rise in Fat Mass on Adipose Tissue Before and During Pregnancy in Rats

**DOI:** 10.3389/fphys.2019.00209

**Published:** 2019-03-06

**Authors:** Layla Albustanji, Gabriela S. Perez, Enas AlHarethi, Peter Aldiss, Ian Bloor, Jairza M. Barreto-Medeiros, Helen Budge, Michael E. Symonds, Neele Dellschaft

**Affiliations:** ^1^Early Life Research Unit, Division of Child Health, Obstetrics, and Gynaecology, University of Nottingham, Nottingham, United Kingdom; ^2^Graduate Program of Food Nutrition and Health, Department of Food Science, School of Nutrition, Federal University of Bahia, Salvador, Brazil; ^3^CAPES Foundation, Ministry of Education of Brazil, Brasília, Brazil; ^4^Nottingham Digestive Disease Centre and Biomedical Research Centre, School of Medicine, University of Nottingham, Nottingham, United Kingdom

**Keywords:** thermogenesis, brown fat, obesity, temperature, pregnancy

## Abstract

**Aim:** To investigate whether housing temperature influences rat adiposity, and the extent it is modified by diet and/or pregnancy. Housing temperature impacts on brown adipose tissue, that possess a unique uncoupling protein (UCP) 1, which, when activated by reduced ambient temperature, enables rapid heat generation.

**Methods:** We, therefore, examined whether the effects of dietary induced rise in fat mass on interscapular brown fat in female rats were dependent on housing temperature, and whether pregnancy further modulates the response. Four week old rats were either maintained at thermoneutrality (27°C) or at a “standard” cool temperature (20°C), and fed either a control or obesogenic (high in fat and sugar) diet until 10 weeks old. They were then either tissue sampled or mated with a male maintained under the same conditions. The remaining dams were tissue sampled at either 10 or 19 days gestation.

**Results:** Diet had the greatest effect on fat mass at thermoneutrality although, by 19 days gestation, fat weight was similar between groups. Prior to mating, the abundance of UCP1 was higher at 20°C, but was similar between groups during pregnancy. *UCP1* mRNA followed a similar pattern, with expression declining to a greater extent in the animals maintained at 20°C.

**Conclusion:** Housing temperature has a marked influence on the effect of dietary induced rise in fat deposition that was modified through gestation. This maybe mediated by the reduction in UCP1 with housing at thermoneutrality prior to pregnancy and could subsequently impact on growth and development of the offspring.

## Introduction

The temperature in which laboratory animals are maintained can have a pronounced impact on both metabolic and physiological homeostasis (Maloney et al., 2014). This is important when investigating adipose tissue function, as brown fat is very sensitive to ambient temperature and its activity is enhanced when rats are kept at temperatures below thermoneutrality, i.e., less than 27°C (Gordon, 1990). Consequently, the metabolic and endocrine effects of dietary induced obesity are likely to be underestimated when animals are maintained at the standard housing temperature of 20°C, which represents a chronic cool challenge (Xiao et al., 2015; Cui et al., 2016). Furthermore, humans usually live in an environment close to their thermoneutral zone, which needs taking into account when relating rodent studies to the human situation (Maloney et al., 2014; Gordon, 2017).

Housing temperature will also be important in the interpretation of studies investigating the impact of maternal obesity on pregnancy outcomes. For example, the majority of rodent studies show little stimulatory effect of obesity on birth weight (Shankar et al., 2007; Morris and Chen, 2009) and some even show an increased incidence of intra-uterine growth retardation (Howie et al., 2009). This is important as the main consequence of maternal obesity in otherwise apparently healthy women is increased birth weight (Ruager-Martin et al., 2010). Before examining the magnitude of postnatal outcome, it is necessary to establish whether ambient temperature has comparable effects in the mother, to those which have been described primarily in male mice (Stemmer et al., 2015; Xiao et al., 2015; Cui et al., 2016).

The extent to which a dietary induced increase in fat mass prior to pregnancy modulates brown adipose tissue (BAT) remains to be established. The primary functional marker of BAT is uncoupling protein (UCP)1 (Aquila et al., 1985; Cannon and Nedergaard, 2004), that is located on the inner mitochondrial membrane. When UCP1 is stimulated, this results in the free flow of protons across the inner mitochondrial membrane (Nicholls, 1983) thereby bypassing the need to convert ADP to ATP as occurs in the mitochondria of all other tissues. In rodents fed a standard diet it has been suggested that the activity of BAT declines with pregnancy to enable the conservation of maternal energy stores (Martin et al., 1989). Not all studies have, however, found this (Frontera et al., 2005). A recent study in mice suggests the activity of BAT may decline between conception and day 14 of gestation (McIlvride et al., 2017). This conclusion was based on “*in vivo*” measurements made in terminally anaesthetized animals and did account for the reduction in BAT function it causes (Ohlson et al., 1994). The same study also suggested that removal of interscapular fat from the mother influenced fetal weight, but considered the fetus rather than the dam, as the unit of experimentation, which is incorrect (Festing, 2006). Pregnancy, and diet could also impact on the abundance of beige adipocytes, although currently temperature is considered to be the primary determinant (Wu et al., 2012).

We examined the impact of housing temperature on UCP1 in the primary BAT depot in rodents (i.e., interscapular) and the extent to which either protein or gene expression could be modulated by being maintained at a “standard” cool temperature (i.e., 20°C) or at thermoneutrality (i.e., ∼27°C). In addition, we investigated whether any such response was modified by dietary induced increase in fat mass. We also determined whether primary gene markers of BAT function and growth [i.e., *UCP1*, *PGC1α*, and *PPARγ* (Puigserver et al., 1998)], plus those involved in fat transport [i.e., *FATP4*, *CD36*, and *LPL* (Jemaa et al., 1995; Herrmann et al., 2001; Le Foll et al., 2015)] and insulin sensitivity [i.e., *IRS1* and *2* (Rondinone et al., 1997)], were modified in pregnancy. Some, but not all, of these genes are temperature sensitive in brown or beige adipocytes (Puigserver et al., 1998; Bartelt et al., 2011; Ye et al., 2013), but have yet to be examined through gestation. The same measurements were also performed in a fat depot considered to exhibit an increase in beige characteristics following cold exposure, i.e., the inguinal depot, as well as one that does not i.e., the omental fat depot (Waldén et al., 2012). We hypothesized that the impact of diet and pregnancy on adipose tissue would be amplified when animals were maintained at thermoneutrality and that this would be mediated by changes in BAT.

## Materials and Methods

### Animals and Diets

A summary of the animal protocol is illustrated in Supplementary Figure S1 and was undertaken under the United Kingdom Animals (Scientific Procedures) Act, 1986 with approval from the Local Ethics Committee of the University of Nottingham (Nottingham, United Kingdom).

Eighty-eight female and 16 male Sprague-Dawley rats were obtained at 4 weeks of age from Charles River Laboratories. Half of the male and female rats were immediately randomized to be housed either at a cool temperature (19–21°C) or at thermoneutrality (26–28°C). They were also randomized to receive either a low fat, low sucrose diet (L; 18% of energy from fat (soybean oil); 24% from protein; 58% from carbohydrates, of which 7% are mono- and disaccharides; 3.1 kcal/g; Harlan Teklad Global 18% Protein Rodent Diet; Teklad Diets, Madison, WI, United States) or a high fat, high sucrose diet (H; 39% of energy from fat (lard); 23% from protein; 39% from carbohydrate, of which 44% are mono- and disaccharides; 4.6 kcal/g; custom diet supplied by Abbott Nutrition, Granada, Spain; Supplementary Table S1), fed *ad libitum*. Food intake was measured over the course of three days before mating occurred. Starting numbers of males and females in the H groups were higher than in the L groups as we anticipated a lower fertility although this did not occur. Twelve females (3 at 20L, 2 at 20H, 2 at 27L, 5 at 27H) had reabsorbed all fetuses or failed to mate and were therefore not analyzed. Final groups were, therefore, 20L (18 females, 3 males), 20H (21 females, 5 males), 27L (19 females, 3 males), 27H (18 females, 5 males). All animals were kept in 12:12 h light:dark cycle at 40–50% humidity, and in groups of 3 females and 2 or 3 males to reduce social stress, but had no observable impact on their behavior.

Body weight was recorded three times a week. At 10 weeks of age, 6 randomly selected females of each group were euthanased and tissues were collected. The remaining females were mated with males of the same diet and temperature group. Vaginal plugs found in the morning indicated day 1 of pregnancy. Females were returned to their home cages and re-housed with the same cage mates, but because of the 4 day reproductive cycle of the rat, each mother did not become pregnant on the same day. This meant it was not possible to accurately measure food intake for each group of mothers through pregnancy. Females were euthanased and tissues were collected from 6 to 7 dams per group at 10 days gestation (d/g), coincident with mid-gestation, and from 6 to 8 dams per group near term i.e., 19 d/g.

### Tissue Collection

Females were not fasted before tissue collection to avoid the fasting effects on brown adipocytes. They were euthanased between 10.00 and 12.00 h by CO_2_ asphyxiation and subsequent cervical dislocation. A blood sample was collected into EDTA tubes after cardiac puncture, and interscapular, inguinal, omental (mesenteric), perirenal and gonadal adipose depots were collected and weighed. Blood was centrifuged at 4°C (15 min at 2000g) and plasma aliquoted and stored at −80°C until analysis. The interscapular adipose depot was halved, with one half snap frozen in liquid nitrogen and stored at −80°C until analysis, and the other half fixed in 4% formaldehyde in 0.9% saline before dehydration and blocking in paraffin. An aliquot of the remaining adipose tissues was snap frozen in liquid nitrogen and stored at −80°C until analysis.

### Plasma Metabolites and Hormones

Plasma was thawed gently on ice. Plasma concentrations of glucose (GAGO-20, Sigma-Aldrich, Gillingham, United Kingdom), triglycerides (GPO DAOS method, Wako, Neuss, Germany), non-esterified fatty acids [NEFA-HR(2), Wako, Neuss, Germany], insulin (80-INSRT-E01, Alpco, Salem, NH, United States), corticosterone (K014-H1, Arbor Assays, Ann Arbor, MI, United States) and leptin (EZRL-83K, Merck, Darmstadt, Germany) were measured with commercial assays, respectively, following manufacturer’s instructions.

### UCP1 Immunohistochemistry

Paraffin-blocked interscapular adipose tissue were sectioned to 6 μm and applied to Superfrost Plus slides (Thermo Fisher Scientific, Wilmingont, DE, United States). Slides were processed in a Bond Max IHC stainer (Leica Biosystems, Milton Keynes, United Kingdom) with rabbit anti-UCP1 antibody (ab10983, Abcam, Cambridge, United Kingdom; 1:3000), and compared to negative control slides without primary antibody applied. Slides were viewed and photographed at 10× magnification (Nikon Eclipse 90i, Nikon, Tokyo, Japan; with Hamamatsu ORCA-ER camera, Hamamatsu, Hamamatsu City, Japan). A minimum of five images per sample were analyzed with Fiji-native methods, (Schindelin et al., 2012) using Weka segmentation (Arganda-Carreras et al., 2017).

### Gene Expression Analysis

Adipose tissues were homogenized using a Dispomix (Wilten, Etten-Leur, Netherlands), and RNA was extracted from 50 to 100 mg of tissue using TRI reagent (Sigma-Aldrich, Gillingham, United Kingdom) and chloroform (Thermo Fisher Scientific, Wilmingont, DE, United States) within the Qiagen RNeasy kit (Hilden, Germany) with gDNA eliminator columns. Concentration and purity of eluted RNA was measured on a Nanodrop spectrometer (Thermo Fisher Scientific, Wilmington, DE, United States). One μg of RNA was reverse transcribed to cDNA with the High Capacity RNA-t-cDNA kit (Applied Biosystems, Thermo Fisher Scientific, Wilmington, DE, United States).

Gene expression was measured using fast SYBR Green (Applied Biosystems, Thermo Fisher Scientific, Wilmington, DE, United States) in StepOnePlus Real-Time PCR system (Applied Biosystems, Thermo Fisher Scientific, Wilmington, DE, United States). Specificity of primers (Sigma-Aldrich, Gillingham, United Kingdom; working concentration 250 nM) was confirmed by sequencing PCR product (primers are listed in Supplementary Table S2). Gene expression was assessed for the following pathways:

(i) Thermogenesis: *UCP1*, peroxisome proliferator activated receptor gamma (*PPARG*), PPARG coactivator 1 alpha (*PGC1a*), leptin, beta-3 adrenergic receptor (*B3AR*), vascular endothelial growth factor A (*VEGFA*), capsaicin receptor (*TRPV1*), hydroxysteroid 11 beta dehydrogenase (*BHSD11*), voltage-dependent anion channel 1 (*VDAC*), ATPase sarcoplasmic/endoplasmic reticulum Ca2+ (*SERCA2B*), ryanodine receptor 2 (*RYR2*);
(ii) Insulin sensitivity and energy sensing: insulin receptor substrates 1 and 2 (*IRS1+2*), mammalian target or rapamycin (*mTOR*), transcription factor 7 like 2 (*TCF7L2*);
(iii) Fat transport: fatty acid transport protein 4 (*FATP4*), fatty acid binding protein 4 (*FABP4*), fatty acid translocase (*CD36*), lipoprotein lipase (*LPL*), adipose triglyceride lipase (*ATGL*);
(iv) Immune response: tumor necrosis factor (*TNF*), interleukin 6 (*IL6*), monocyte chemotactic protein 1 (*MCP1*), adhesion G protein-coupled receptor E1 (*EMR1*);

Gene expression was normalized to the geometric mean of housekeeping genes TATA-box binding protein (*TBP*) and tyrosine 3-monooxygenase/tryptophan 5-monooxygenase activation protein (*YWHAZ*) throughout, and was calculated using the 2-ΔΔCt method (Livak and Schmittgen, 2001).

### Statistical Analysis

All statistical analyses were carried out in SPSS (Version 22, IBM, Portsmouth, United Kingdom) and Graphpad (Version 6, GraphPad Software, La Jolla, CA, United States). Data was tested for normality using a Kolmogorov-Smirnov test. If normally distributed, data was analyzed (i) with two-way ANOVAs with LSD *post hoc* comparisons (20L vs. 20H, 27L vs. 27H, 20L vs. 27L, and 20H vs. 27H) within each of the three time points (0, 10, and 19 d/g) to test for changes due to the interventions, and (ii) with one-way ANOVAs with *a priori* comparisons (0 vs. 10 or 19, and 10 vs. 19 d/g) within each interventional group to test for changes with pregnancy. If data was not normally distributed, Kruskal-Wallis tests with equivalent Mann-Whitney tests were carried out.

## Results

### Body Weight and Fat Mass

**FIGURE 1:**
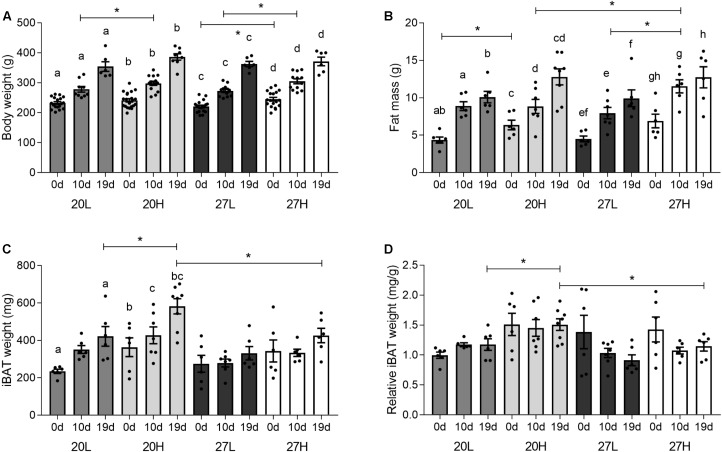
Effect of housing temperature, pregnancy, and diet on body and fat mass. **(A)** body weight, **(B)** fat mass, expressed as the total sum of dissected inguinal, perirenal, omental, and gonadal adipose tissue, **(C)** total, and **(D)** relative interscapular brown fat mass of females before (0 days) and during pregnancy (10 and 19 days), when kept at either a cool temperature (20°C) or thermoneutrality (27°C), and fed a low (L) or high (H) fat and sucrose diet. Fat mass was also expressed as change from unmated **(C)**. Differences between dietary groups housed at the same temperature ^∗^*P* < 0.05; for each study group columns with the same superscripts are significantly different (*P* < 0.05). For body weight, unmated *n* = 18–21, 10 d/g *n* = 10–14, 19 d/g *n* = 6–8; for fat mass, unmated *n* = 6, 10 d/g *n* = 6–7, 19 d/g *n* = 6–8.

Before mating, body weight was only higher due to the H diet when animals were kept at thermoneutrality, whereas fat mass was higher due to the H diet only at the low temperature (Figure 1). Body weight and fat mass increased with pregnancy in all interventional groups. By 10 d/g, body weight was higher with the H diet regardless of temperature, whereas fat mass was only raised in those dams maintained at thermoneutrality. Body weight and fat mass were similar between groups by 19 d/g. Fetal weight was not different between any of the groups, at either sampling gestations and increased with gestation e.g., 20H: 88 ± 2 mg at 10 d/g; 135 ± 3 mg at 19 d/g.

### Metabolic and Endocrine Profiles

Before mating, plasma glucose concentrations were notably higher in animals maintained at thermoneutrality but were similar between groups through pregnancy (Table 1). Plasma triglyceride and leptin increased with gestation in all groups, whereas NEFA concentrations were only increased at 19 d/g in the 20H group. Insulin was raised with the H diet before mating, but this only reached statistical significance for animals raised at 20°C. Although insulin concentrations were similar between groups through pregnancy there was high variability. Corticosterone was similar between groups, although mean values were highest in the 27L group, this difference was not significant. It was low in the 27L group at 10 d/g to increase by 19 d/g. Food intake, measured just before mating, was higher in the H diet group only when animals were kept at thermoneutrality when consumption of the L diet was reduced [20L: 57 ± 3; 20H 67 ± 3; 27L: 45 ± 3; 27H: 66 ± 5 kcal/d (*P* < 0.05)].

**Table 1:** Plasma metabolites hormones of females before and during pregnancy, when kept at either a cool temperature (20°C) or thermoneutrality (27°C), and fed a low (L) or high (H) fat and sucrose diet.

Plasma measurement	Pregnancy time point (d/g)	20L	20H	27L	27H	*ANOVA*
Glucose (μg ml^−1^)	0	24 ± 4^ac^	28 ± 5	47 ± 6^c^	40 ± 4	*0.009*
	10	48 ± 9^ab^	38 ± 6	43 ± 9	35 ± 3	*N.S.*
	19	22 ± 2^b^	35 ± 9	28 ± 4	30 ± 4	*N.S.*
	*ANOVA*	*0.008*	*N.S.*	*N.S.*	*N.S.*	
Triglycerides (mg dl^−1^)	0	95 ± 14^a^	118 ± 19^c^	82 ± 17^e^	127 ± 28^g^	*N.S.*
	10	106 ± 19^ab^	107 ± 14^cd^	130 ± 20^ef^	143 ± 27^gh^	*N.S.*
	19	245 ± 35^b^	300 ± 36^d^	320 ± 58^f^	227 ± 26^h^	*N.S.*
	*ANOVA*	*0.008*	*0.006*	*0.002*	*0.0387*	
NEFA (mmol l^−1^)	0	0.17 ± 0.02	0.24 ± 0.05	0.31 ± 0.06	0.21 ± 0.03	*N.S.*
	10	0.15 ± 0.02	0.17 ± 0.02^a^	0.15 ± 0.01	0.20 ± 0.03	*N.S.*
	19	0.22 ± 0.03	0.33 ± 0.02^a^	0.24 ± 0.04	0.31 ± 0.04	*N.S.*
	*ANOVA*	*N.S.*	*0.010*	*N.S.*	*N.S.*	
Insulin (ng ml^−1^)	0	0.95 ± 0.12^a^	1.77 ± 0.21^a^	0.97 ± 0.14	1.67 ± 0.62	*0.040*
	10	1.24 ± 0.35	0.96 ± 0.10	1.17 ± 0.24	1.92 ± 0.23	*N.S.*
	19	1.24 ± 0.30	1.84 ± 0.45	3.58 ± 1.12	1.53 ± 0.46	*N.S.*
	*ANOVA*	*N.S.*	*N.S.*	*N.S.*	*N.S.*	
Corticosterone (ng ml^−1^)	0	169 ± 52	106 ± 35	450 ± 171	107 ± 23	*N.S.*
	10	68 ± 20	180 ± 100	32 ± 7^a^	148 ± 94	*N.S.*
	19	111 ± 18	159 ± 25	242 ± 112^a^	178 ± 46	*N.S.*
	*ANOVA*	*N.S.*	*N.S.*	*0.016*	*N.S.*	
Leptin (ng ml^−1^)	0	3.2 ± 0.4^ab^	4.7 ± 0.6^c^	3.4 ± 0.8^de^	4.4 ± 0.2^fg^	*N.S.*
	10	5.5 ± 0.6^a^	7.1 ± 1.1	5.6 ± 0.7^d^	11.0 ± 2.1^f^	*N.S.*
	19	6.9 ± 0.7^b^	7.8 ± 1.2^c^	8.2 ± 1.7^e^	9.1 ± 1.6^g^	*N.S.*
	*ANOVA*	*0.002*	*0.047*	*0.029*	*0.023*	

*P values given are the statistically significant outcomes of comparisons (ANOVA or Kruskal-Wallis, indicated as “ANOVA”). If data within one outcome carry the same superscripts they are statistically different (P < 0.05). For all plasma measurements, unmated n = 5–6, 10 d/g n = 6–7, 19 d/g n = 5–8.*

### Interscapular Adipose Depot Weight, UCP1 Protein, and Gene Expression

Interscapular adipose mass was similar between unmated animals (Figure 1C,D), but during pregnancy it was lower in animals at thermoneutrality, in which there was a smaller rise with gestation compared with those kept at a 20°C. Additionally, at 19 d/g, 20H animals had a greater interscapular fat mass than 20L, whereas diet had no effect at thermoneutrality.

**FIGURE 2:**
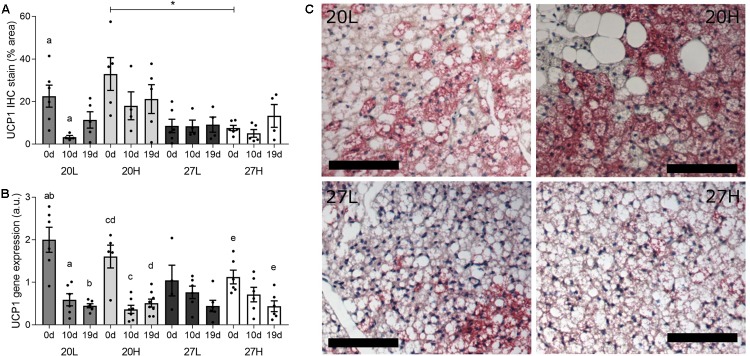
Effect of housing temperature, pregnancy and diet on uncoupling protein (UCP)1. **(A)** (UCP)1 protein, **(B)** *UCP1* mRNA abundance, and **(C)** representative histological images of interscapular adipose tissue of females before (0 d/g) and during pregnancy (10 and 19 d/g), when kept at either a cool temperature (20°C) or thermoneutrality (27°C), and fed a low (L) or high (H) fat and sucrose diet. Differences between dietary groups housed at the same temperature ^∗^*P* < 0.05; for each study group columns with the same superscripts are significantly different (*P* < 0.05). For interscapular adipose tissue mass, unmated *n* = 6, 10 d/g *n* = 6–7, 19 d/g *n* = 6–8; for UCP1 protein, unmated *n* = 5–6, 10 d/g *n* = 4–6, 19 d/g *n* = 4–6. Representative anti-UCP1 immunohistochemistry images of interscapular adipose tissue **(C)** from unmated females of the 20L (top left), 20H (top right), 27L (bottom left), and 27H groups (bottom right), with size bars indicating 100 μm.

**FIGURE 3:**
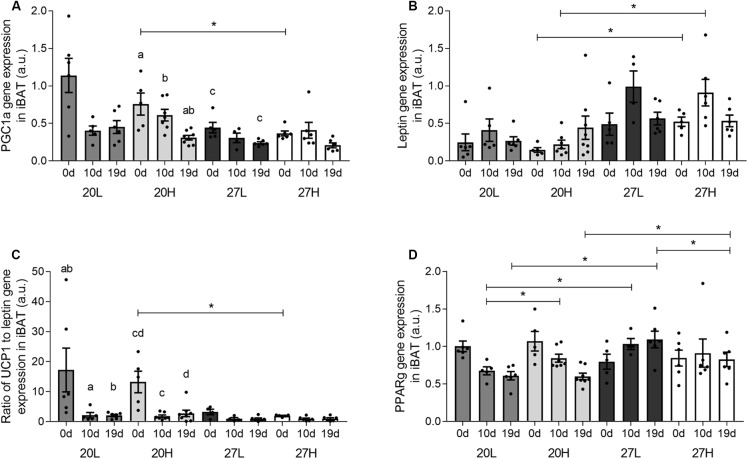
Effect of housing temperature, pregnancy, and diet on interscapular gene expression. **(A)** *PGC1a*, **(B)** *leptin*, **(C)** the ratio of *UCP1* to *leptin* gene expression, and **(D)** *PPARG* of females before (0 days) and during pregnancy (10 and 19 days), when kept at either a cool temperature (20°C) or thermoneutrality (27°C), and fed a low (L) or high (H) fat and sucrose diet. Differences between dietary groups housed at the same temperature ^∗^*P* < 0.05; for each study group columns with the same superscripts are significantly different (*P* < 0.05). For all interscapular gene expression, unmated *n* = 5–6, 10 d/g *n* = 5–7, 19 d/g *n* = 6–8.

Uncoupling protein 1 protein was more abundant in unmated animals fed the H diet at 20°C than at thermoneutrality (Figure 2). In contrast, *UCP1* gene expression was similar between groups and declined with gestation, and was accompanied with similar changes in *PGC1α* (Figure 3). There was also a higher gene expression of *PGC1α* in 20H compared with 27H groups prior to mating. Gene expression of leptin, a marker of white fat, was higher before, and in mid-pregnancy, in the 27H, compared with the 20H group. When comparing the ratio of *UCP1* and *leptin* gene expression, i.e., the main genes characteristic of brown and white adipocytes, respectively, the ratio of gene expression was markedly higher in unmated animals at 20°C, and then declined to similar mean values for all groups through pregnancy. *PPARγ* gene expression also declined with pregnancy in the animals kept at 20°C, irrespective of diet.

Other thermogenic genes examined i.e., *B3AR* and *SERCA2B* were unaffected by pregnancy, diet, or temperature, whilst *TRPV1* gene expression was higher at 10 than 19 d/g (Table 2). Gene expression of *BHSD11* was consistently higher in 27H than 20H groups and higher in 27L than 20L at 19 d/g, as it rose substantially by the end of pregnancy in those dams maintained at thermoneutrality. Energy sensing genes i.e., *TCFL2* and *mTOR* were consistently upregulated through pregnancy but were not affected by diet or temperature. *IRS1* gene expression was lower at thermoneutrality at 19 d/g regardless of diet, and was downregulated with pregnancy in the 27H group, whilst *IRS2* was reduced with the H diet prior to pregnancy. Subsequently, there was no difference between groups as mRNA abundance increased up to 10 d/g and was similar between all groups by term. Of the genes involved in fat transport, *CD36* showed an increase in late pregnancy, but only at thermoneutrality. *ATGL* expression was higher in 27L than 27H, a difference that was not present at 20°C, as it was downregulated with pregnancy in the 20L group. At both environmental housing temperatures, *LPL* peaked at 10 d/g in those dams fed the control diet. *FATP4* gene expression increased with pregnancy regardless of any intervention.

**Table 2:** Interscapular gene expression measurements of genes of thermogenic, energy regulatory, and fat transport pathways of females before and during pregnancy, when kept at either a cool temperature (20°C) or thermoneutrality (27°C), and fed a low (L) or high (H) fat and sucrose diet.

Gene	Pregnancy time point (d/g)	20L	20H	27L	27H	*ANOVA*
**Thermogenesis**						
B3AR	0	0.89 ± 0.06	0.84 ± 0.06^a^	0.90 ± 0.15	0.71 ± 0.10	*N.S.*
	10	0.62 ± 0.09	0.46 ± 0.06^ab^	0.67 ± 0.05	0.64 ± 0.11	*N.S.*
	19	0.73 ± 0.08	0.65 ± 0.08^b^	0.73 ± 0.13	0.47 ± 0.06	*N.S.*
	*ANOVA*	*N.S.*	*0.006*	*N.S.*	*N.S.*	
SERCA2B	0	0.49 ± 0.18	0.58 ± 0.16^a^	0.41 ± 0.09	0.43 ± 0.12	*N.S.*
	10	0.32 ± 0.17	0.20 ± 0.16^ab^	0.17 ± 0.07	0.31 ± 0.14	*N.S.*
	19	0.32 ± 0.12	0.56 ± 0.17^b^	0.48 ± 0.16	0.28 ± 0.10	*N.S.*
	*ANOVA*	*N.S.*	*0.028*	*N.S.*	*N.S.*	
TRPV1	0	0.58 ± 0.09^a^	0.57 ± 0.06^c^	0.75 ± 0.16	0.86 ± 0.18^f^	*N.S.*
	10	1.73 ± 0.39^ab^	1.75 ± 0.26^cd^	1.62 ± 0.54^e^	1.17 ± 0.13^g^	*N.S.*
	19	0.61 ± 0.05^b^	0.72 ± 0.16^d^	0.45 ± 0.05^e^	0.46 ± 0.06^fg^	*N.S.*
	*ANOVA*	*0.011*	*0.006*	*0.013*	*0.004*	
BHSD11	0	0.41 ± 0.05^ab^	0.30 ± 0.05^de^	0.64 ± 0.13^bi^	0.64 ± 0.05^ek^	*0.020*
	10	0.61 ± 0.26	0.27 ± 0.05^fg^	0.50 ± 0.09^j^	0.53 ± 0.05^gl^	*0.014*
	19	0.73 ± 0.07^ac^	0.92 ± 0.13^dfh^	2.80 ± 0.23^cij^	3.11 ± 0.51^hkl^	*<0.001*
	*ANOVA*	*0.032*	*0.001*	*0.003*	*0.003*	
**Insulin sensitivity and energy sensing**						
TCF7L2	0	0.65 ± 0.04^ab^	0.70 ± 0.07^c^	0.70 ± 0.09^d^	0.67 ± 0.11^e^	*N.S.*
	10	1.24 ± 0.20^a^	1.14 ± 0.11^c^	0.95 ± 0.12	0.80 ± 0.13	*N.S.*
	19	1.62 ± 0.14^b^	1.53 ± 0.15^c^	1.33 ± 0.19^d^	2.80 ± 1.06^e^	*N.S.*
	*ANOVA*	*0.001*	*0.001*	*0.020*	*0.030*	
mTOR	0	0.27 ± 0.03^a^	0.28 ± 0.02^b^	0.26 ± 0.03^c^	0.24 ± 0.03^d^	*N.S.*
	10	0.91 ± 0.06^a^	1.00 ± 0.11^b^	1.08 ± 0.08^c^	0.85 ± 0.07^de^	*N.S.*
	19	0.55 ± 0.04^a^	0.43 ± 0.03^b^	0.43 ± 0.06^c^	0.34 ± 0.06^e^	*N.S.*
	*ANOVA*	*<0.001*	*<0.001*	*0.002*	*0.003*	
IRS1	0	0.92 ± 0.14	0.81 ± 0.04	0.66 ± 0.14	0.66 ± 0.06^d^	*N.S.*
	10	1.00 ± 0.11	0.86 ± 0.09	0.75 ± 0.12	0.76 ± 0.04^e^	*N.S.*
	19	0.67 ± 0.05^a^	0.65 ± 0.05^b^	0.49 ± 0.07^ac^	0.31 ± 0.05^bcde^	*0.001*
	*ANOVA*	*N.S.*	*N.S.*	*N.S.*	*<0.001*	
IRS2	0	0.096 ± 0.014^ab^	0.052 ± 0.004^bd^	0.124 ± 0.020^fg^	0.055 ± 0.012^fi^	*0.008*
	10	0.069 ± 0.019^c^	0.058 ± 0.008^e^	0.088 ± 0.024^h^	0.058 ± 0.006^j^	*N.S.*
	19	1.01 ± 0.16^ac^	0.98 ± 0.14^de^	1.24 ± 0.21^fh^	0.97 ± 0.23^ij^	*N.S.*
	*ANOVA*	*0.002*	*0.001*	*0.003*	*0.006*	
**Fat transport**						
CD36	0	0.98 ± 0.10	0.92 ± 0.10	0.83 ± 0.04^c^	0.80 ± 0.08^e^	*N.S.*
	10	0.67 ± 0.09	0.78 ± 0.10	0.84 ± 0.07^d^	0.89 ± 0.07^f^	*N.S.*
	19	0.85 ± 0.12^a^	1.00 ± 0.04^b^	1.16 ± 0.06^acd^	1.12 ± 0.04^bef^	*0.006*
	*ANOVA*	*N.S.*	*N.S.*	*0.001*	*0.012*	
ATGL	0	1.40 ± 0.15^ab^	1.14 ± 0.04	1.28 ± 0.14^d^	0.85 ± 0.11^d^	*0.020*
	10	0.90 ± 0.14^a^	0.83 ± 0.14	1.15 ± 0.09	1.07 ± 0.12	*N.S.*
	19	0.91 ± 0.05^bc^	0.90 ± 0.07	1.51 ± 0.12^ce^	1.03 ± 0.12^e^	*<0.001*
	*ANOVA*	*0.015*	*N.S.*	*N.S.*	*N.S.*	
LPL	0	0.91 ± 0.08^a^	0.86 ± 0.07	0.79 ± 0.07^c^	0.64 ± 0.07	*N.S.*
	10	1.03 ± 0.04^b^	0.89 ± 0.07	1.05 ± 0.10^cd^	0.72 ± 0.07^d^	*0.016*
	19	0.59 ± 0.04^ab^	0.68 ± 0.05	0.53 ± 0.06^c^	0.53 ± 0.07	*N.S.*
	*ANOVA*	*<0.001*	*N.S.*	*0.001*	*N.S.*	
FATP4	0	0.59 ± 0.04^ab^	0.56 ± 0.03	0.62 ± 0.05^cd^	0.59 ± 0.04^ef^	*N.S.*
	10	0.92 ± 0.06^a^	0.86 ± 0.05	1.00 ± 0.05^c^	0.89 ± 0.04^e^	*N.S.*
	19	0.85 ± 0.10^b^	0.87 ± 0.14	1.32 ± 0.15^d^	1.17 ± 0.15^f^	*N.S.*
	*ANOVA*	*0.016*	*N.S.*	*0.001*	*0.002*	

*P values given are the statistically significant outcomes of comparisons (ANOVA or Kruskal-Wallis, indicated as “ANOVA”). If data within one outcome carry the same superscripts they are statistically different (P < 0.05).For all interscapular gene expression, unmated n = 5–6, 10 d/g n = 5–7, 19 d/g n = 6–8. B3AR, beta-3 adrenergic receptor; SERCA2B, ATPase sarcoplasmic/endoplasmic reticulum Ca2+; TRPV1, capsaicin receptor; BHSD11, hydroxysteroid 11 beta dehydrogenase; TCF7L2, transcription factor 7 like 2; mTOR, mammalian target or rapamycin; IRS1+2, insulin receptor substrates 1 and 2; CD36, fatty acid translocase; ATGL, adipose triglyceride lipase; LPL, lipoprotein lipase; FATP4, fatty acid transport protein 4.*

### Inguinal and Omental Adipose Depots

*UCP1* mRNA was undetectable in the inguinal (or omental) depot. *PPARG* and *IRS2* gene expression declined through gestation in all groups (Table 3), whilst *B3AR* was undetectable at the end of pregnancy. *Leptin* gene expression declined by mid gestation in the 20L group only, whilst *FABP4* only decreased with gestation in the 27L group.

**Table 3:** Inguinal adipose tissue gene expression measurements of genes of thermogenic, energy regulatory and fat transport pathways of females before and during pregnancy, when kept at either a cool temperature (20°C) or thermoneutrality (27°C), and fed a low (L) or high (H) fat and sucrose diet.

Gene	Pregnancy time point (d/g)	20L	20H	27L	27H	*ANOVA*
**Thermogenesis**						
PPARG	0	1.02 ± 0.15^a^	0.71 ± 0.09^b^	0.84 ± 0.12^d^	0.89 ± 0.16^d^	*N.S.*
	10	0.78 ± 0.11	0.59 ± 0.13^c^	0.72 ± 0.04^e^	0.76 ± 0.12^e^	*N.S.*
	19	0.50 ± 0.07^a^	0.29 ± 0.05^bc^	0.46 ± 0.08^de^	0.39 ± 0.04^de^	*N.S.*
	*ANOVA*	*0.020*	*0.009*	*0.016*	*0.025*	
B3AR	0	0.75 ± 0.14^a^	0.59 ± 0.09	0.91 ± 0.13	0.79 ± 0.13	*N.S.*
	10	0.30 ± 0.06^a^	0.42 ± 0.10	0.57 ± 0.10	0.63 ± 0.12	*N.S.*
	19	N.D.	N.D.	N.D.	N.D.	*N.S.*
	*ANOVA*	*0.020*	*N.S.*	*N.S.*	*N.S.*	
LEPTIN	0	0.50 ± 0.16^a^	0.31 ± 0.09	0.47 ± 0.13	0.20 ± 0.05	*N.S.*
	10	0.11 ± 0.03^ab^	0.13 ± 0.02	0.23 ± 0.05	0.19 ± 0.03	*N.S.*
	19	0.26 ± 0.04^b^	0.20 ± 0.05	0.21 ± 0.06	0.25 ± 0.04	*N.S.*
	*ANOVA*	*0.021*	*N.S.*	*N.S.*	*N.S.*	
**Insulin sensitivity and energy sensing**						
IRS2	0	0.45 ± 0.04^a^	0.47 ± 0.08^b^	0.56 ± 0.03^d^	0.55 ± 0.19^e^	*N.S.*
	10	0.21 ± 0.03^a^	0.32 ± 0.06^c^	0.26 ± 0.04^d^	0.17 ± 0.02^ef^	*N.S.*
	19	1.26 ± 0.20^a^	1.32 ± 0.16^bc^	1.23 ± 0.21^d^	1.07 ± 0.10^f^	*N.S.*
	*ANOVA*	*0.001*	*0.001*	*<0.001*	*0.002*	
**Fat transport**						
FABP4	0	0.59 ± 0.20	0.37 ± 0.07	0.69 ± 0.22^a^	0.38 ± 0.06	*N.S.*
	10	0.18 ± 0.04	0.18 ± 0.04	0.27 ± 0.05^ab^	0.28 ± 0.05	*N.S.*
	19	0.29 ± 0.03	0.20 ± 0.03	0.30 ± 0.05^b^	0.29 ± 0.04	*N.S.*
	*ANOVA*	*N.S.*	*N.S.*	*0.023*	*N.S.*	

*P values given are the statistically significant outcomes of comparisons (ANOVA or Kruskal-Wallis, indicated as “ANOVA”). If data within one outcome carry the same superscripts they are statistically different (P < 0.05). For all inguinal AT gene expression, unmated n = 6, 10 d/g n = 5–7, 19 d/g n = 6–8. PPARG, peroxisome proliferator activated receptor gamma; B3AR, beta-3 adrenergic receptor; IRS2, insulin receptor substrate 2; FABP4, fatty acid binding protein 4.*

There were few differences in gene expression between groups in omental fat (Table 4). In contrast to the inguinal depot, *B3AR* was expressed throughout pregnancy, though declined by 19 d/g. *RYR2* gene expression was also higher in unmated animals and declined with pregnancy in all groups except 20L. Gene expression of *IRS2* was high before mating in the animals kept at thermoneutrality and declined during pregnancy. *FATP4* gene expression increased at 10 d/g but then decreased by 19 d/g, a pattern that is also seen for *FABP4* in 27L but no other groups. Expression of genes of the immune response pathways were not affected, with the exception of a reduction in *MCP1* through pregnancy.

**Table 4:** Omental adipose tissue gene expression measurements of genes of thermogenic, energy regulatory, fat transport, and immune response pathways of females before and during pregnancy, when kept at either a cool temperature (20°C) or thermoneutrality (27°C), and fed a low (L) or high (H) fat and sucrose diet.

Gene	Pregnancy time point (d/g)	20L	20H	27L	27H	*ANOVA*
**Thermogenesis**						
B3AR	0	0.23 ± 0.12	0.62 ± 0.22	0.47 ± 0.14	0.99 ± 0.27	*N.S.*
	10	0.78 ± 0.37	1.46 ± 0.51	1.37 ± 0.34^a^	1.00 ± 0.29	*N.S.*
	19	0.45 ± 0.11	0.40 ± 0.15	0.32 ± 0.14^a^	0.33 ± 0.06	*N.S.*
	*ANOVA*	*N.S.*	*N.S.*	*0.028*	*N.S*.	
RYR2	0	1.17 ± 0.38	2.16 ± 0.46^a^	1.92 ± 0.49^b^	2.34 ± 0.56^d^	*N.S.*
	10	0.83 ± 0.15	1.09 ± 0.20	1.09 ± 0.22^c^	0.85 ± 0.19	*N.S.*
	19	0.73 ± 0.18	0.61 ± 0.10^a^	0.40 ± 0.09^bc^	0.50 ± 0.11^d^	*N.S.*
	*ANOVA*	*N.S.*	*0.017*	*0.007*	*0.021*	
LEPTIN	0	0.22 ± 0.15	0.38 ± 0.14	0.24 ± 0.16	0.65 ± 0.13	*N.S.*
	10	0.56 ± 0.23	0.62 ± 0.19	0.60 ± 0.16	0.95 ± 0.28	*N.S.*
	19	0.50 ± 0.13	0.42 ± 0.13	0.46 ± 0.21	0.53 ± 0.19	*N.S.*
	*ANOVA*	*N.S.*	*N.S.*	*N.S.*	*N.S.*	
TRPV1	0	1.62 ± 1.10	2.45 ± 1.08	0.91 ± 0.25^a^	2.65 ± 0.89	*N.S.*
	10	2.25 ± 0.84	2.29 ± 0.95	3.78 ± 0.58^a^	3.17 ± 0.93	*N.S.*
	19	4.67 ± 0.85	2.56 ± 0.56	2.22 ± 0.68	3.18 ± 0.85	*N.S.*
	*ANOVA*	*N.S.*	*N.S.*	*0.012*	*N.S.*	
VEGFA	0	1.24 ± 0.61	1.62 ± 0.63	1.08 ± 0.22	2.22 ± 0.59^a^	*N.S.*
	10	0.84 ± 0.35	1.10 ± 0.33	1.91 ± 0.68	0.86 ± 0.23	*N.S.*
	19	0.94 ± 0.20	0.88 ± 0.19	0.60 ± 0.18	0.61 ± 0.07^a^	*N.S.*
	*ANOVA*	*N.S.*	*N.S.*	*N.S.*	*0.048*	
SERCA2B	0	0.23 ± 0.10	0.62 ± 0.21	0.68 ± 0.37	1.38 ± 0.45	*N.S.*
	10	0.28 ± 0.11	0.76 ± 0.31	0.62 ± 0.12	0.60 ± 0.29	*N.S.*
	19	0.51 ± 0.14	0.32 ± 0.10	0.30 ± 0.10	0.45 ± 0.17	*N.S.*
	*ANOVA*	*N.S.*	*N.S.*	*N.S.*	*N.S.*	
VDAC	0	2.03 ± 0.90	3.43 ± 1.05	1.86 ± 0.44	3.34 ± 0.85	*N.S.*
	10	3.01 ± 0.94	2.95 ± 0.92	4.67 ± 0.83	4.50 ± 1.28	*N.S.*
	19	4.68 ± 0.73	3.37 ± 0.70	2.88 ± 0.77	3.40 ± 0.77	*N.S.*
	*ANOVA*	*N.S.*	*N.S.*	*N.S.*	*N.S.*	
**Insulin sensitivity and energy sensing**						
IRS1	0	0.38 ± 0.13	0.62 ± 0.24	0.68 ± 0.24	1.44 ± 0.56	*N.S.*
	10	0.67 ± 0.26	0.84 ± 0.25	0.99 ± 0.20	0.57 ± 0.16	*N.S.*
	19	0.76 ± 0.16	0.61 ± 0.15	0.52 ± 0.23	0.51 ± 0.10	*N.S.*
	*ANOVA*	*N.S.*	*N.S.*	*N.S.*	*N.S.*	
IRS2	0	1.02 ± 0.23	0.56 ± 0.23	2.01 ± 0.40^ab^	1.97 ± 0.61^c^	*N.S.*
	10	0.91 ± 0.27	0.78 ± 0.17	0.78 ± 0.17^a^	0.64 ± 0.15	*N.S.*
	19	0.73 ± 0.16	0.89 ± 0.14	0.63 ± 0.13^b^	0.57 ± 0.10^c^	*N.S.*
	*ANOVA*	*N.S.*	*N.S.*	*0.007*	*0.047*	
**Fat transport**						
FATP4	0	0.23 ± 0.04	0.19 ± 0.03	0.23 ± 0.02^b^	0.27 ± 0.02^d^	*N.S.*
	10	0.35 ± 0.07^a^	0.30 ± 0.05	0.75 ± 0.34^bc^	1.51 ± 0.87^de^	*N.S.*
	19	0.15 ± 0.01^a^	0.20 ± 0.03	0.17 ± 0.03^c^	0.14 ± 0.02^e^	*N.S.*
	*ANOVA*	*0.023*	*N.S.*	*0.018*	*0.015*	
FABP4	0	0.48 ± 0.30	1.35 ± 0.52	0.46 ± 0.16^a^	1.01 ± 0.19	*N.S.*
	10	0.51 ± 0.20	1.38 ± 0.49	1.91 ± 0.20^ab^	1.48 ± 0.42	*N.S.*
	19	1.18 ± 0.23	0.67 ± 0.23	0.62 ± 0.21^b^	0.89 ± 0.26	*N.S.*
	*ANOVA*	*N.S.*	*N.S.*	*<0.001*	*N.S.*	
**Immune response**						
EMR1	0	0.72 ± 0.16	0.72 ± 0.16	0.61 ± 0.09	0.82 ± 0.15^ab^	*N.S.*
	10	0.79 ± 0.25	0.67 ± 0.17	0.85 ± 0.15	0.37 ± 0.10^a^	*N.S.*
	19	0.64 ± 0.06	0.79 ± 0.20	0.50 ± 0.14	0.40 ± 0.05^b^	*N.S.*
	*ANOVA*	*N.S.*	*N.S.*	*N.S.*	*0.016*	
TNF	0	1.26 ± 0.24	0.77 ± 0.25	1.11 ± 0.33	0.65 ± 0.20	*N.S.*
	10	0.90 ± 0.24	0.59 ± 0.17	0.42 ± 0.11	0.50 ± 0.22	*N.S.*
	19	0.44 ± 0.14	1.09 ± 0.31	0.76 ± 0.12	0.59 ± 0.19	*N.S.*
	*ANOVA*	*N.S.*	*N.S.*	*N.S.*	*N.S.*	
IL6	0	0.036 ± 0.013^ab^	0.024 ± 0.006	0.019 ± 0.005	0.012 ± 0.002	*N.S.*
	10	0.010 ± 0.003^a^	0.012 ± 0.003	0.010 ± 0.002	0.009 ± 0.002	*N.S.*
	19	0.012 ± 0.003^b^	0.016 ± 0.004	0.014 ± 0.003	0.015 ± 0.006	*N.S.*
	*ANOVA*	*0.017*	*N.S.*	*N.S.*	*N.S.*	
MCP1	0	2.76 ± 0.92^a^	4.80 ± 1.04^ef^	2.93 ± 0.30^gh^	2.85 ± 0.70^ij^	*N.S.*
	10	0.47 ± 0.06^a^	0.57 ± 0.07^e^	0.59 ± 0.07^g^	0.43 ± 0.09^i^	*N.S.*
	19	1.12 ± 0.14^abcd^	0.66 ± 0.06^bf^	0.71 ± 0.09^ch^	0.51 ± 0.05^dj^	*0.005*
	*ANOVA*	*0.003*	*0.002*	*0.002*	*0.012*	

*P values given are the statistically significant outcomes of comparisons (ANOVA or Kruskal-Wallis, indicated as “ANOVA”). If data within one outcome carry the same superscripts they are statistically different (P < 0.05). For all omental AT gene expression, unmated n = 5–6, 10 d/g n = 5–7, 19 d/g n = 6–8. B3AR, beta-3 adrenergic receptor; RYR2, ryanodine receptor 2; TRPV1, capsaicin receptor; VEGFA, vascular endothelial growth factor A; SERCA2B, ATPase sarcoplasmic/endoplasmic reticulum Ca2+; VDAC, voltage-dependent anion channel 1; IRS1+2, insulin receptor substrates 1 and 2; FATP4, fatty acid transport protein 4; FABP4, fatty acid binding protein 4; EMR1, adhesion G protein-coupled receptor E1; TNF, tumor necrosis factor; IL6, interleukin 6; MCP1, monocyte chemotactic protein 1.*

## Discussion

This is the first study to investigate the impact of housing temperature on a dietary induced rise in fat mas followed by pregnancy and further demonstrates its impact on metabolic outcomes (Maloney et al., 2014; Gordon, 2017). Ambient temperature modulated the effect of a diet that persisted into pregnancy. This could be explained, in part, by reduced UCP1 protein and increased food intake before pregnancy. Irrespective of diet, unmated females maintained at thermoneutrality had raised glucose, that could be mediated by a lower rate of thermogenesis, especially in BAT (Zingaretti et al., 2009). However, the effect of temperature on plasma glucose did not persist into pregnancy, possibly due to the concomitant metabolic adaptations (Bell and Bauman, 1997), and/or due to changes in UCP1. These findings complement the effect of housing at thermoneutrality demonstrated in male mice, in which UCP1 abundance is reduced, whereas effects on glucose, and insulin diminish with time (Xiao et al., 2015; Small et al., 2018). Moreover, the raised maternal glucose at the time of mating could impact on embryo development without an immediate impact on fetal growth (Lewis et al., 2013). Ideally, we would also have made additional measurements on UCP1 but with the limited amount of tissue available meant we could only undertake measures of gene expression and histology.

Overall, there was a decrease in *UCP1* and *PGC1α* gene expression within BAT through gestation which was more pronounced at 20°C than at thermoneutrality. No other gene examined showed a comparable response, which was accompanied with a transient decline in UCP1 protein at 20°C, but not thermoneutrality. A recent study in mice (McIlvride et al., 2017) suggested that UCP1 protein decreased with gestation, although this was based on a single immunohistochemistry slide, without quantification. The same study also suggested that the thermogenic response was reduced with gestation, but this was measured in terminally anaesthetized animals, that suppresses BAT function (Ohlson et al., 1994) and potentially confounding effects of this procedure in pregnancy cannot be ruled out. A further report suggested UCP1 protein declines with pregnancy in mice (Qiao et al., 2018). These measurements were, however, made in total protein homogenates, rather than mitochondrial preparations which is where UCP1 resides (Aquila et al., 1985) and is known to decline with gestation (Frontera et al., 2005). In the present study, the lack of any quantitative, sustained change in UCP1 protein with gestation is indicative of maintained function, given it is the protein and not mRNA that determines heat production from UCP1 (Nedergaard and Cannon, 2013).

We observed modest temperature dependent changes in gene expression of some BAT markers by late gestation e.g., a decline in *PPARγ* was only seen at 20°C, whilst *CD36* and *BHSD11* were raised considerably more substantially at thermoneutrality. It is likely that the maternal endocrine adaptations with gestation mediate these responses and could include changes in the prolactin-growth hormone axis as well as steroid hormones (Napso et al., 2018). The largest relative change in gene expression with gestation within BAT we observed was for *leptin*, and this was confined to those dams kept at thermoneutrality. The extent to which such an adaptation could impact on BAT function has yet to be established, although recent *in vitro* studies suggest an increase in leptin co-localisation within the nucleus that co-regulates the appearance of UCP1 with cold exposure (Velickovic et al., 2018). Interestingly, the increase in *leptin* gene expression was confined to BAT and was not seen within the inguinal fat depot that did not express *UCP1*. As inguinal fat has been considered to be an important beige depot, the absence of *UCP1* gene expression suggests such adaptations may be confined to males, or that lower ambient temperatures are required in females (Waldén et al., 2012). Neither the inguinal and omental fat depots expressed UCP1 mRNA and were both unresponsive to changes in housing temperature. Taken together these findings emphasize the importance of the presence of UCP1 in modulating the effect of temperature on adipose function.

In conclusion, housing temperature determines the effect of dietary induced obesity on fat deposition, although this response was modified through gestation. This adaptation maybe mediated by reduced UCP1 content of brown fat seen with the H diet prior to pregnancy.

## Data Availability

All datasets generated for this study are included in the manuscript and/or the Supplementary Files.

## Author Contributions

MS, HB, and ND conceived and designed the study, interpreted the data, refined the manuscript, and critiqued the output for intellectual content. MS, ND, LA, and GP conducted the animal study. LA, GP, EA, IB, PA, and ND undertook the laboratory analyses. LA and GP equally wrote the first manuscript draft. All authors reviewed the final manuscript.

## Conflict of Interest Statement

The authors declare that the research was conducted in the absence of any commercial or financial relationships that could be construed as a potential conflict of interest.
