# Supplementary material for: Housing Temperature Modulates the Impact of Diet-Induced Rise in Fat Mass on Adipose Tissue Before and During Pregnancy in Rats
Source: Front Physiol. 2019 Mar 6;10:209. doi: 10.3389/fphys.2019.00209 (PMC6414463; doi:10.3389/fphys.2019.00209)
Supplement: Supplementary file 1 [file Data_Sheet_1.docx]

**Supplementary Figure 1**: Schematic of the study design. Female and male Sprague Dawley rats were kept at either standard housing temperature or at thermoneutrality and on either a high fat, high sucrose diet (HFHS) or low fat, low sucrose diet (LFLS) for six weeks before mating. Females were sacrificed at 10 weeks of age (n=6), and after mating at 10d (n=6-7) or 19d gestation (n=6-8). Females which failed to mate or lost pregnancy were not included in the n-numbers shown.


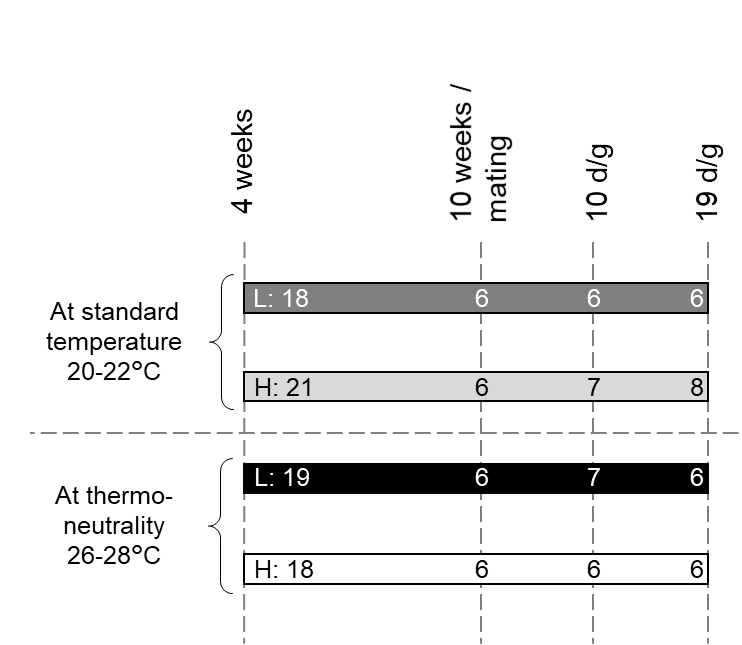


**Supplementary Table 1**: Macronutrient composition of diets.

|  | Low fat, low sucrose diet (L) | High fat, high sucrose diet (H) |
| --- | --- | --- |
| *% of weight* | | |
| Protein | 18.6 | 26.2 |
| Fat | 6.2 | 20 |
| Carbohydrate | 44.2 | 44.7 |
| Mono-, disaccharides | 3 | 19.5 |
| Energy density (kcal g-1) | 3.1 | 4.6 |
| *% of energy* | | |
| Protein | 24.2 | 22.6 |
| Fat | 18.2 | 38.8 |
| Carbohydrate | 57.6 | 38.6 |
| Mono-, disaccharides | 3.9 | 16.8 |

**Supplementary Table 2**: Primers used for measurements of gene expression. For each gene, the nucleotide sequence of the primer pair is stated, together with the length of the amplicon (in base pairs) and accession number.

| **Pathway** | **Gene** | **Sequence (5'->3')** | **Product length** | **Accession number** |
| --- | --- | --- | --- | --- |
| Thermogenesis | UCP1 | GCCTAGCAGACATCATCACCT | 147 | NM_012682.2 |
|  |  | GTTTCGGCAATCCTTCTGTC |  |  |
|  | PPARG | CCCAATGGTTGCTGATTACA | 76 | XM_006237009.3 |
|  |  | GGACGCAGGCTCTACTTTGA |  |  |
|  | PGC1a | Bio-Rad probe qRnoCIP0022855 | 102 | NM_031347 |
|  | Leptin | TTCACACACGCAGTCGGTAT | 186 | XM_008762762.2 |
|  |  | AGGTCTCGCAGGTTCTCCAG |  |  |
|  | B3AR | TAGCAAGGAGCCTGACTTCTG | 133 | NM_013108.2 |
|  |  | TTCTGGAGAGTTGCGGTTCC |  |  |
|  | VEGFA | TGAGTTAAACGAACGTACTTGCAG | 91 | NM_001287113.1 |
|  |  | TCTAGTTCCCGAAACCCTGA |  |  |
|  | TRPV1 | CATGGGTGAGACCGTCAACA | 116 | NM_031982.1 |
|  |  | AGGCCTTCCTCATGCACTTC |  |  |
|  | BHSD11 | Bio-Rad probe qRnoCEP0023342 | 103 | NM_017080 |
|  | VDAC | GTTGGGGATGCGAGAGTTGA | 105 | NM_031353.1 |
|  |  | GGAATGGGGTTTCCGCTGTA |  |  |
|  | SERCA2B | TCATGGACGAGACGCTCAAG | 163 | NM_001110139.2 |
|  |  | TTCCCCAAGCTCAGTCATGC |  |  |
|  | RYR2 | GGAAGTGAAGCAGCCCAAG | 71 | NM_001191043.1 |
|  |  | TCATCCATGTGTCCATGTAGC |  |  |
| Insulin sensitivity and energy sensing | IRS-1 | GAAGTTCCTTCCGCAGTGTC | 155 | NM_012969.1 |
|  |  | GTTGCCACCCCTAGACAAAA |  |  |
|  | IRS-2 | CTACCCACTGAGCCCAAGAG | 151 | NM_001168633.1 |
|  |  | CCAGGGATGAAGCAGGACTA |  |  |
|  | mTOR | TGGAGGGAGAGCGTCTGAGA | 111 | NM_019906.1 |
|  |  | TGATGTGCCGAGGCTTTGT |  |  |
|  | TCF7L2 | GTCCACCCACTCACACCTCT | 100 | NM_001191052.1 |
|  |  | TTCCTGTTTTGGGGTCTACG |  |  |
| Fat transport | FATP-4 | GACTTCTCCAGCCGTTTCCA | 93 | NM_001100706.1 |
|  |  | CACCTGGCTGTCAAAGTTGC |  |  |
|  | FABP-4 | AACTGGGCGTGGAATTCGAT | 147 | NM_053365.1 |
|  |  | CCAGCTTGTCACCATCTCGT |  |  |
|  | CD36 | GCAGCCTCCTTTCCACCTTT | 176 | NM_001109218.1 |
|  |  | TTGTCTGGGTTCTGGAGTGG |  |  |
|  | LPL | TGGTGGGAAATGATGTGGCC | 188 | NM_012598.2 |
|  |  | TGCATACTCAAAGTTAGGCCCA |  |  |
|  | ATGL | Bio-Rad probe qRnoCEP0023736 | 101 | NM_001108509 |
| Immune response | TNF | ACGTCGTAGCAAACCACCAA | 164 | XM_008772775.2 |
|  |  | GCAGCCTTGTCCCTTGAAGA |  |  |
|  | IL-6 | ATGGATGCTTCCAAACTGGAT | 139 | NM_012589.2 |
|  |  | TGAATGACTCTGGCTTTGTCT |  |  |
|  | MCP1 | AGCATCCACGTGCTGTCTC | 78 | NM_031530.1 |
|  |  | GATCATCTTGCCAGTGAATGAG |  |  |
|  | EMR1 | AATTTCACAGACCAAGAAGTGACA | 111 | XM_006244280.3 |
|  |  | GCAGTTGTAGGAACCTGGTACA |  |  |
| Housekeeping Genes | YWHAZ | GATGAAGCCATTGCTGAACTTG | 117 | XM_006241529.3 |
|  |  | GTCTCCTTGGGTATCCGATGTC |  |  |
|  | TBP | TGGGATTGTACCACAGCTCCA | 132 | XM_006227980.3 |
|  |  | CTCATGATGACTGCAGCAAACC |  |  |

Thermogenesis: uncoupling protein 1 (*UCP1*), peroxisome proliferator activated receptor gamma (*PPARG*), PPARG coactivator 1 alpha (*PGC1a*), leptin, beta-3 adrenergic receptor (*B3AR*), vascular endothelial growth factor A (*VEGFA*), capsaicin receptor (*TRPV1*), hydroxysteroid 11 beta dehydrogenase (*BHSD11*), voltage-dependent anion channel 1 (*VDAC*), ATPase sarcoplasmic/endoplasmic reticulum Ca^2+^ (*SERCA2B*), ryanodine receptor 2 (*RYR2*);

Insulin sensitivity and energy sensing: insulin receptor substrates 1 and 2 (*IRS1+2*), mammalian target or rapamycin (*mTOR*), transcription factor 7 like 2 (*TCF7L2*);

Fat transport: fatty acid transport protein 4 (*FATP4*), fatty acid binding protein 4 (*FABP4*), fatty acid translocase (*CD36*), lipoprotein lipase (*LPL*), adipose triglyceride lipase (*ATGL*);

Immune response: tumour necrosis factor (*TNF*), interleukin 6 (*IL6*), monocyte chemotactic protein 1 (*MCP1*), adhesion G protein-coupled receptor E1 (*EMR1*);

Housekeeping genes: TATA-box binding protein (*TBP*), Tyrosine 3-monooxygenase / tryptophan 5-monooxygenase activation protein (*YWHAZ*).
